# Ethanol Extracts of *Solanum lyratum Thunb* Regulate Ovarian Cancer Cell Proliferation, Apoptosis, and Epithelial-to-Mesenchymal Transition (EMT) via the ROS-Mediated p53 Pathway

**DOI:** 10.1155/2021/5569354

**Published:** 2021-04-01

**Authors:** Chen Zhang, Zheming Li, Jie Wang, Xuelu Jiang, Mengting Xia, Jianfen Wang, Shenyi Lu, Shouye Li, Hanmei Wang

**Affiliations:** ^1^Department of Obstetrics and Gynecology, The First Affiliated Hospital of Zhejiang Chinese Medical University (Zhejiang Provincial Hospital of Traditional Chinese Medicine), Hangzhou City 310001, China; ^2^College of Pharmacy, Hangzhou Medical College, Hangzhou City 310053, China; ^3^Department of Obstetrics and Gynecology, Zhejiang Chinese Medical University, Hangzhou City 310001, China; ^4^Department of Obstetrics and Gynecology, Zhuji Hospital Affiliated to Shaoxing University, Zhuji City 311800, China

## Abstract

Ovarian cancer is a type of common gynecological tumors with high incidence and poor survival. The anticancer effects of the traditional Chinese medicine *Solanum lyratum Thunb* (SLT) have been intensively investigated in various cancers but in ovarian cancer is rare. The current study is aimed at investigating the effect of SLT on ovarian cancer cells. Lactate dehydrogenase (LDH) and MTT assays indicated that SLT concentrations of 0.25 and 0.5 *μ*g/mL were not cytotoxic and had significant inhibitory effects on the cell viabilities of A2780 and SKOV3 cells, hence were used for subsequent experiments. Flow cytometric and western blot analysis revealed that SLT effectively suppressed ovarian cancer cell proliferation via inducing cell cycle arrest and increasing apoptosis. Cell cycle and apoptosis-related protein expressions were also regulated in SLT-treated cells. Moreover, DCFH-DA and western blot assays demonstrated that SLT enhanced ROS accumulation and subsequently activated the p53 signaling pathway. However, SLT-regulated ovarian cancer cell proliferation, apoptosis, migration, invasion, and EMT were significantly reversed by an ROS inhibitor (NAC, N-acetyl-L-cysteine). Furthermore, A2780 and SKOV3 cells cocultured with M0 macrophages showed that SLT activated the polarization of M0 macrophages to M1 macrophages and inhibited the polarization to M2 macrophages, with the increased percentage of CD86+ cells and decreased percentage of CD206+ cells were detected. In summary, this study illustrated the anticancer effects of SLT on ovarian cancer cells, suggesting that SLT may have the potential to provide basic evidence for the discovery of antiovarian cancer agents.

## 1. Introduction

Human ovarian cancer is the most fatal cancer in the female reproductive system [1–3]. The traditional treatment process is to remove the tumor to the maximum and then combine the paclitaxel and platinum chemotherapy [4]. Although 70% of the patients initially respond well to the chemotherapy, while the emergence of drug resistance and side effects curb the benefits of currently available chemotherapy drugs [5], and the 5-year survival is still unsatisfactory [6–8]. Since few of these strategies can completely protect patients with ovarian cancer from recurrence and metastasis, new drugs are urgently needed. One way to solve this problem is to develop new drugs that are used in combination with existing chemotherapy drugs to produce better results than simple chemotherapy.

In terms of adjuvant therapy, the efficacy and safety of traditional Chinese medicine (TCM) in the management of cancers have gained increasing acceptance worldwide; TCM were also usually served as rich resources for drug discovery of pharmaceutical companies [9, 10]. In addition to the price advantage, TCM treatment has been reported to have the advantages of chemotherapy efficacy strengthening, toxicity reduction, survival time prolongation, and improving the quality of life and immune function [11, 12]. There are increasing evidences that many natural products, including extracts and isolated chemicals, may interact with multiple targets in signaling pathways that regulate cancer progression [9]. Therefore, it is necessary to study the natural products systematically, clarify their antitumor effects, and understand their mechanism, so as to develop new therapeutic methods.

*Solanum lyratum Thunb* (SLT) is a common TCM, which is widely used in China to treat malaria, jaundice, cholecystitis, gonorrhea, rheumatoid arthritis, leucorrhea, and cancer [13]. Since the 20th century, a variety of active compounds have been identified in SLT, including saponins, lignans, alkaloids, polyphenols, flavonoids, and polysaccharides [14, 15]. A series of evidences have shown that SLT has antitumor activity, thus attracting much attention. In lung carcinoma-bearing mouse model, study of SLT showed antitumor effect and can improve immune function and survival rate of mice [16]. SLT extracts can induce cell cycle arrest and apoptosis in human osteosarcoma U-2 OS cells, and this worked through the ROS-promoted and mitochondria- and caspase-dependent apoptotic pathways [17]. Another study indicated that the extract of SLT induced cytotoxicity and apoptosis of human colon cancer cell line Colo 205, which may be associated with CDK1, p27, p53, cyclin B1, cyclin E, and apoptosis-related proteins, as well as the activity of cytochrome C [18]. Solasodine, an alkaloid compound extracted from SLT, has been reported to regulate cell apoptosis and autophagy and reduce the metastasis of ovarian cancer cells [19]. These researches suggest that SLT has the potential to be an adjuvant drug for the treatment of cancers. However, the effects of SLT extract on ovarian cancer and its mechanism remain unclear. In the present study, the effects of SLT on the proliferation, apoptosis, epithelial-to-mesenchymal transformation (EMT), and immunomodulatory potential of ovarian cancer cells were detected, and hypothesis of the antiovarian cancer effect was possibly through the regulation of the p53 pathway mediated by ROS.

## 2. Materials and Methods

### 2.1. Cell Culture

Human ovarian cancer cell lines, A2780 and SKOV3 cells, were obtained from American Type Culture Collection (ATCC, Rockville, MD, USA) and cultured in Dulbecco's modified Eagle medium (DMEM, Invitrogen, Carlsbad, CA, USA) supplemented with 10% fetal bovine serum (FBS, Sigma, St. Louis, MO, USA) and 100 U/mL penicillin and 100 *μ*g/mL streptomycin (Invitrogen) in 5% CO_2_ at 37°C. Human monocytes such as THP-1 were obtained from the Cellular Library of the National Collection of Authenticated Cell Cultures (Shanghai, China) and cultured in RPMI 1640 medium supplemented with 10% FBS in 5% CO_2_ at 37°C.

### 2.2. Preparation of SLT Extract

50 g SLT was crushed and immersed in 75% ethanol for 3 h, and then soaked and filtered again for three times. The SLT extract was then obtained by mixing the filtrate and drying it with a rotary evaporator. Different concentrations of SLT preparation using dimethyl sulfoxide (DMSO, Sigma-Aldrich, Saint Louis, MO, USA) dissolve SLT extract in preparation of the drug solution concentration of 10 mg/mL, and then diluted with cell culture medium to obtain the concentrations of 0, 0.1, 0.25, 0.5, 1.0, 2.5, 5, and 10 *μ*g/mL SLT extract solution. Taxol (Sigma-Aldrich) dissolved in DMSO and diluted with cell culture medium to a final concentration of 1 *μ*M was used as the positive control.

### 2.3. MTT Assay

Cells were seeded into 96-well plates at a cell density of 4 × 10^3^ cells per well, cultured overnight, and treated with different concentrations of SLT extract solution or taxol solution for 24 or 48 h. 20 *μ*L of 5 mg/mL MTT solution (5 mg/mL, Sigma) was added into each well for another 4 h of incubation; thereafter, the suspension was removed, and the precipitate was dissolved in 100 *μ*L DMSO. Absorbance levels were measured at 570 nm using a SpectraMax M5 microplate reader (Molecular Devices, Sunnyvale, CA, USA). The experiments were performed in triplicate.

### 2.4. Lactate Dehydrogenase (LDH) Assay

Cells were seeded into 96-well plates at a density of 4 × 10^3^ cells per well, cultured overnight, and treated with different concentrations of SLT extract solution or taxol solution for 48 h. LDH release was detected by LDH cytotoxicity assay kit (Beyotime, Nantong, Jiangsu, China) based on the manufacturer's instructions. Absorbance levels were measured at 490 nm using a SpectraMax M5 microplate reader.

### 2.5. Cell Cycle Assay

Cells were collected after 48 h of exposure to SLT, rinsed thrice with PBS, harvested, and fixed in 70% ice-cold ethanol at −4°C overnight. Subsequently, 50 ng/mL propidium iodide (Beyotime) staining solution and 0.1 mg/mL RNase A were added to the cells away from the light for 30 min at room temperature. The cell cycle distribution of cells was analyzed using a flow cytometer (BD Biosciences, Franklin Lakes, NJ, USA).

### 2.6. Wound Healing Assay

Cells were seeded into 6-well plates and maintained overnight. When cells reached 90% confluence, they were treated with 10 *μ*g/mL mitomycin C for 2 h as described in the literature. The surface layer cells were scratched with a 100 *μ*L sterilized pipette tip on the bottom of each plate after cells reaching 100% confluence. Then, the cell debris was washed, and1 mL fresh serum-free medium was added with SLT at the indicated concentration for 24 h. 0 hours and 24 hours after scratching, use a microscope (Olympus, Tokyo, Japan) to photograph the width of the gap area.

### 2.7. Transwell Assay

Cells (5 × 10^4^ cells/well) pretreated with SLT were seeded in the top chamber (Millipore, Billerica, MA), which was precoated with Matrigel (BD Biosciences, MA). Serum-free culture medium was added to the top chambers, while the bottom chambers were filled with 500 *μ*L cultured medium containing 10% FBS. After incubation for 24 h, cells in the bottom chamber were fixed with 4% paraformaldehyde (PFA), stained with 0.1% crystal violet, and counted under a microscope (Olympus, Tokyo, Japan).

### 2.8. Annexin V-FITC Apoptosis Assay

Cells were harvested and washed thrice with PBS after 48 h of exposure to SLT. Then, cells were incubated with 500 *μ*L of binding buffer containing 5 *μ*L of fluorescein isothiocyanate- (FITC-) labeled Annexin V (Beyotime) and 5 *μ*L of propidium iodide (PI) solution (Beyotime) away from the light for 15 min at room temperature. Then, cell apoptosis rates were analyzed using a flow cytometer (BD Biosciences).

### 2.9. Measurement of Intracellular Reactive Oxygen Species (ROS)

The intracellular levels of ROS were measured using the green fluorescent probe 6-carboxy-2′,7′-dichlorofluoroscein diacetates (DCFH-DA, Sigma-Aldrich, MO, USA). 5 × 10^3^ cells per well were seeded into a 24-well culture plate and exposed to different concentrations of SLT treatment for 6 h, and then stained with 10 *μ*M DCFH-DA for 30 min under dark at 37°C. After washing with PBS twice and fixing with 4% paraformaldehyde, the stained cells were observed under a fluorescence microscope. Fluorescence was measured with microplate spectrofluorometer (Synergy 4, BioTek, VT, USA) with 488 nm excitation wavelength and 525 nm emission wavelength. To inhibit ROS, 1 mM N-acetyl-L-cysteine (NAC) was added into the medium for 2 h. The ratio of average fluorescence intensity of each group to that of the control group represents ROS production.

### 2.10. M0 Macrophage Inducement and Coculture

M0 macrophages were induced from the human monocytes such as THP-1. Briefly, THP-1 cells were seeded in 6-well plates and added with 100 ng/mL phorbol 12-myristate 13-acetate (PMA) in Transwell inserts for 48 h to induce into M0 macrophages. Then, the PMA containing media were removed gently and the activated THP-1 cells were placed into a new 6-well plate adding with 2 mL of fresh RPIM/FCS medium. The differentiated M0-type macrophages were subsequently cocultured with untreated A2780/SKOV-3 cells or A2780/SKOV-3 supplemented with SLT (0.25 and 0.5 *μ*g/mL) to assess the effect of SLT on M1 and M2 polarization of M0 macrophages.

### 2.11. Flow Cytometry

Flow cytometry assay was performed to detect the expression of CD86 for M1 macrophages and CD206 for M2 macrophages. A2780 or SKOV-3 cells were cocultured with M0-type macrophages, and SLT was coadded at different concentrations (0, 0.25, and 0.5 *μ*g/mL). Then, cell suspension was incubated with FITC, PE-, and allophycocyanin- (APC-) conjugated Abs anti-human CD206 and CD86 (BD Biosciences Pharmingen, USA) for 30 min under the dark; the control isotype corresponding to each primary antibody was also added. Then, cells were washed with PBS and analyzed by flow cytometry (BD, USA).

### 2.12. Western Blotting

Proteins were extracted using RIPA lysis (Beyotime, Jiangsu, China) buffer and were collected. Then, protein concentration was determined using a BCA assay kit (Thermo Scientific, Waltham, MA, USA). The protein samples were separated by using sodium dodecyl sulfate-polyacrylamide gel electrophoresis (SDS-PAGE) and electrophoretically transferred onto polyvinylidene fluoride (PVDF, EMD Millipore, Billerica, MA, USA) membranes. Membranes were blocked with 5% skim milk for 1 hour and then incubated with primary antibodies against CDK4 (ab199728, 1 : 2000, Abcam), Cyclin D1 (ab226977, 1 : 500, Abcam), p53 (ab131442, 1 : 1000, Abcam), p21 (ab7960, 1 : 2000, Abcam), MMP2 (ab2462, 1 : 5000, Abcam), MMP9 (ab76003, 1 : 2000, Abcam), E-cadherin (ab18203, 1 : 1000, Abcam), N-cadherin (ab18203, 1 : 1000, Abcam), cleaved caspase-3 (ab2302,1 : 1000, Abcam), cleaved PARP (ab32064, 1 : 1000, Abcam), Bcl-2 (ab32124, 1 : 1000, Abcam), Bax (ab7977, 1 : 1000, Abcam), CD86 (ab7977, 1 : 1000, Abcam), CD206 (ab7977, 1 : 1000, Abcam), iNOS (ab7977, 1 : 1000, Abcam), Arg-1 (ab7977, 1 : 1000, Abcam), and GAPDH (ab8245, 1 : 1000, Abcam) at 4°C for overnight. Subsequently, the membranes were probed with horseradish peroxidase- (HRP-) conjugated secondary antibodies (Abcam) at room temperature for 1 hour. The band was visualized using an enhanced chemiluminescence technique (Thermo Fisher Scientific).

### 2.13. Statistical Analysis

Numerical data are expressed as the mean ± standard deviation (SD) and were analyzed using SPSS 21.0 (Chicago, IL, USA). Statistical analysis between two and multiple groups was performed using Student's *t*-test and one-way analysis of variance(ANOVA) followed by the least significant difference post hoc test. Each experiment was performed independently at least for three times. Differences with values of *P* < 0.05 were considered as statistically significant.

## 3. Results

### 3.1. Effects of SLT on Ovarian Cancer Cell Viability

**Figure 1:**
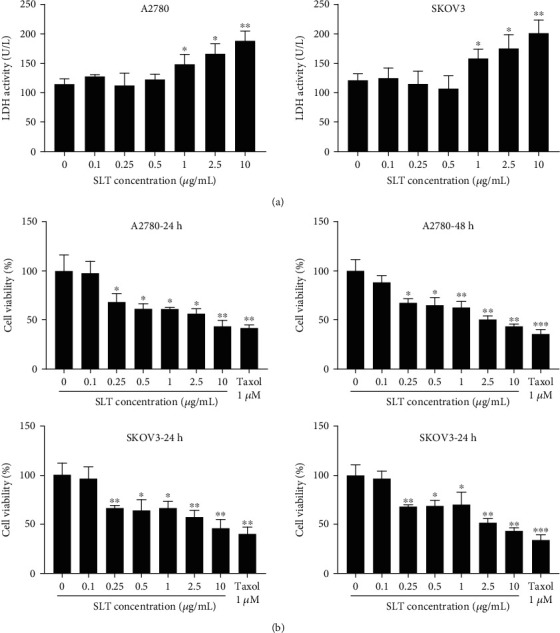
The effects of SLT on ovarian cancer cell viability. (a) The effects of SLT at indicated concentrations on LDH release in A2780 and SKOV3 cells were determined by LDH assay. (b) The effects of SLT at indicated concentrations on the cell viability of A2780 and SKOV3 cells were analyzed by MTT assay. The values were displayed as the mean ± SD. Representative results from three independent experiments were shown (^∗^*P* < 0.05, ^∗∗^*P* < 0.01, and ^∗∗∗^*P* < 0.001, compared with the control group).

Since the concentration of LDH released into the extracellular environment corresponds to the degree of cell membrane damage, we used the LDH release assay to determine the cytotoxicity of SLT (Figure 1(a)). The results demonstrated that over 1.0 *μ*g/mL of SLT remarkably increased LDH leakage of A2780 and SKOV3 cells, which suggested that high concentration of SLT might be cytotoxic. In addition, MTT assay was used to detect the effect of SLT on cell viabilities (Figure 1(b)). The data indicated that the cell viabilities of A2780 and SKOV3 were significantly inhibited by SLT (0.25, 0.5, 1.0, 2.5, and 10 *μ*g/mL). Therefore, SLT concentrations of 0.25 and 0.5 *μ*g/mL were selected in the following experiments.

### 3.2. Effects of SLT on Cell Cycle of Ovarian Cancer

**Figure 2:**
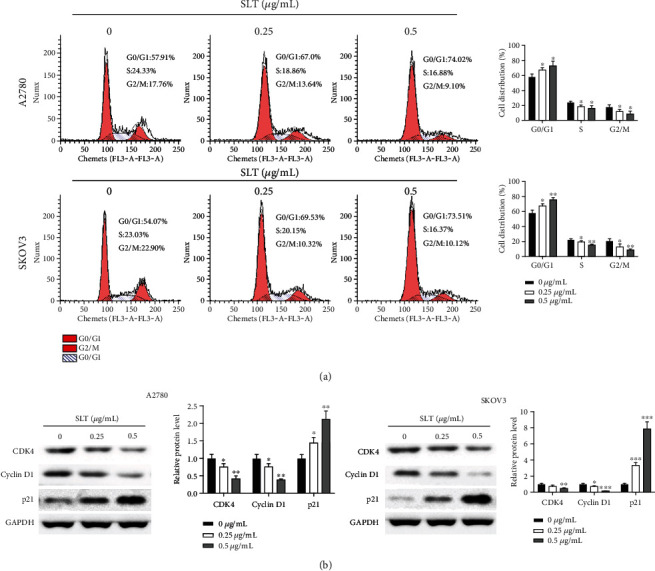
The effects of SLT on cell cycle of ovarian cancer. (a) The effects of SLT on cell cycle distribution of A2780 and SKOV3 cells were analyzed via flow cytometry. (b) The effects of SLT on the expression levels of cell cycle-related proteins in A2780 and SKOV3 cells were evaluated via western blot assay. The values were displayed as the mean ± SD. Representative results from three independent experiments were shown (^∗^*P* < 0.05, ^∗∗^*P* < 0.01, and ^∗∗∗^*P* < 0.001, compared with the control group).

Flow cytometry was used to detect the influence of SLT treatment on cell cycle distribution of A2780 and SKOV3 cells. As represented in Figure 2(a), the proportions of G1 phase A2780 and SKOV3 cells in the SLT treatment (0.25 and 0.5 *μ*g/mL) groups were increased compared with the control group. In addition, SLT treatment (0.25 and 0.5 mg/mL) resulted in a decrease in the percentage of G2/M cells compared with the untreated cells (the control group). SLT significantly increased G1 phase cells accompanied by a decrease in G2/M phase cells, in a concentration-dependent manner, suggesting that SLT blocks cell cycle in G1 phase. Further western blot assay was used to evaluate the effects of SLT on cell cycle regulation-associated proteins. Consistently, SLT treatment significantly reduced the protein levels of CDK4 and Cyclin D1, but increased the protein level of P21 in both A2780 and SKOV3 cells compared to the control group (Figure 2(b)). These data indicated that the inhibitory activity of SLT on A2780 and SKOV3 cell proliferation may be associated with cell cycle arrest at the G1 phase.

### 3.3. Effects of SLT on Apoptosis of Ovarian Cancer Cells

**Figure 3:**
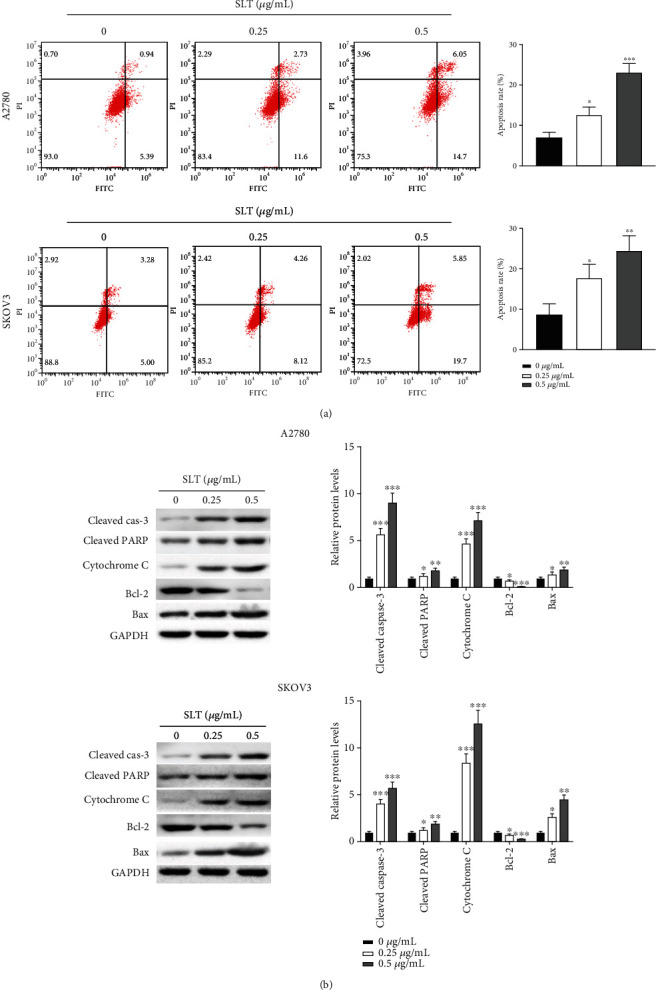
The effects of SLT on apoptosis of ovarian cancer cells. (a) The effects of SLT on cell apoptosis of A2780 and SKOV3 cells were analyzed via Annexin V-FITC/PI double staining assay. (b) The effects of SLT on the expression levels of apoptosis-related protein in A2780 and SKOV3 cells were evaluated via western blot assay. The values were displayed as the mean ± SD. Representative results from three independent experiments were shown (^∗^*P* < 0.05, ^∗∗^*P* < 0.01, and ^∗∗∗^*P* < 0.001, compared with the control group).

Annexin V-FITC/PI double staining assay was used to evaluate the ratio of cell apoptosis after SLT treatment. Results indicated that A2780 and SKOV3 cells after treatment with SLT resulted in a dose-dependent increase of apoptotic cells compared with the control group (Figure 3(a)). The mitochondrial apoptosis pathway plays a key role in the apoptosis signal transduction pathway; hence, we detected changes in apoptosis-related protein levels after SLT treatment. As revealed in Figure 3(b), the protein levels of cleaved caspase-3, cleaved PARP, Bax, and cytochrome C (in the cytoplasm) were significantly increased, and the Bcl-2 level was decreased in the A2780 and SKOV3 cells with SLT treatment compared to the control group (Figure 3(b)). These data suggested that SLT could induce the apoptosis of A2780 and SKOV3 cells which may be partly mediated via a mitochondria-dependent mechanism.

### 3.4. Effects of SLT on Migration, Invasion, and EMT of Ovarian Cancer Cells

**Figure 4:**
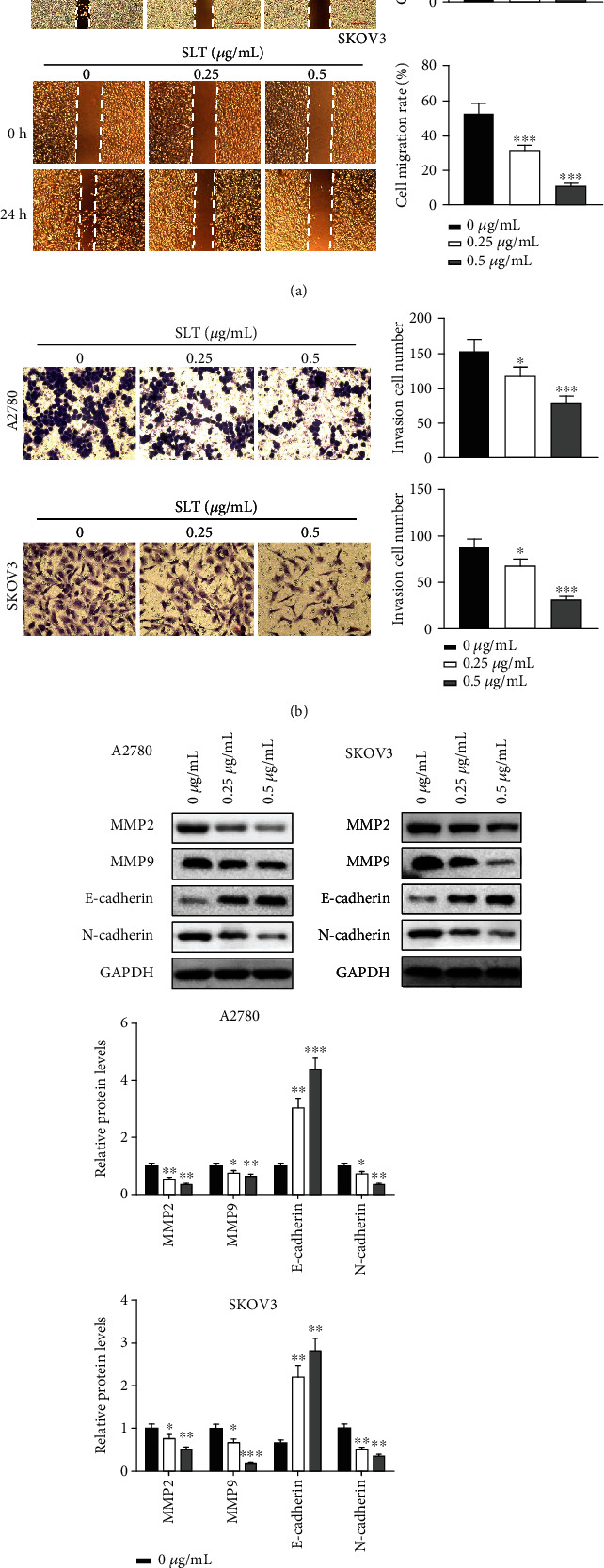
The effects of SLT on migration, invasion, and EMT of ovarian cancer cells. (a) The effects of SLT on cell migration were detected by wound healing assay in A2780 and SKOV3 cells (magnification ×20; scale bar = 1000 *μ*m). (b) The effects of SLT on cell invasion were evaluated by Transwell assay (magnification ×100; scale bar = 200 *μ*m). (c) The effects of SLT on the expression levels of EMT-related protein in A2780 and SKOV3 cells were evaluated via western blot assay. The values were displayed as the mean ± SD. Representative results from three independent experiments were shown (^∗^*P* < 0.05, ^∗∗^*P* < 0.01, and ^∗∗∗^*P* < 0.001, compared with the control group).

The abilities of migration and invasion of A2780 and SKOV3 cells after treatment with SLT were further determined using wound healing and Transwell assays, respectively. As illustrated in Figure 4(a), A2780 and SKOV3 cells in the SLT treatment group had larger wound areas after 24 hours, indicating that cell migration was significantly decreased compared to the control group. With increasing concentration of the SLT (0.25 and 0.5 *μ*g/mL), the number of invaded cells was significantly reduced as compared to the control group, as shown in Figure 4(b). Further western blot assay results (Figure 4(c)) showed that SLT significantly inhibited the protein levels of MMP2, MMP9, and N-cadherin, and increased the protein level of E-cadherin in A2780 and SKOV3 cells. These results demonstrated that SLT reduces the migration, invasion, and EMT of ovarian cancer cells.

### 3.5. Effects of SLT on ROS Production in Ovarian Cancer Cells

**Figure 5:**
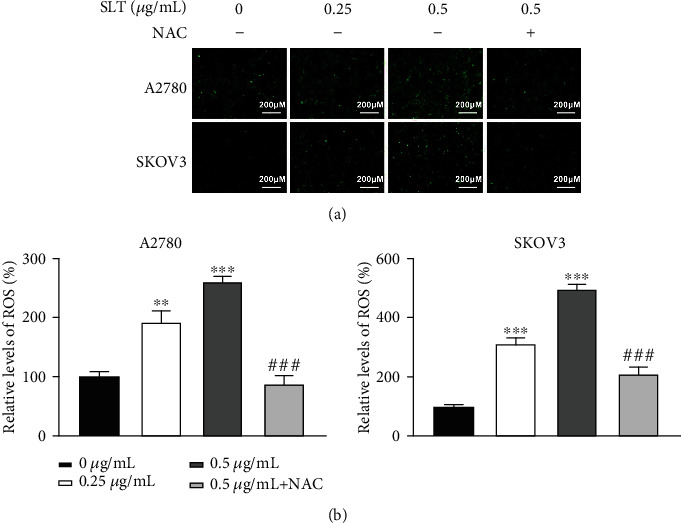
The effects of SLT on ROS production in ovarian cancer cells. (a) The effects of SLT on ROS levels of A2780 and SKOV3 cells were determined using DCF fluorescence by a fluorescence microscope (magnification ×100; scale bar = 40 *μ*m). (b) The DCF fluorescence intensity in A2780 and SKOV3 cells affected by SLT was measured by a fluorescence spectrophotometer. The values were displayed as the mean ± SD. Representative results from three independent experiments were shown (^∗∗^*P* < 0.01 and ^∗∗∗^*P* < 0.001, compared with the control group; ^###^*P* < 0.001, compared with the 0.5 *μ*g/mL SLT group).

As ROS can induce caspase-independent cell death, we detected the SLT-induced ROS production in A2780 and SKOV3 cells. As illustrated in Figures 5(a) and 5(b), significant increases in intracellular ROS levels in A2780 and SKOV3 cells exposed to SLT (0.25 and 0.5 *μ*g/mL) were observed by a fluorescence microscope compared to the control cells. Moreover, in this experiment, A2780 and SKOV3 cells were pretreated with N-acetyl-L-cysteine (NAC), an inhibitor of ROS generation, and then exposed to SLT. And the results indicated that NAC could partially prevent SLT from inducing the production of ROS. All these data suggested that SLT could promote ROS production in ovarian cancer cells.

### 3.6. SLT Induces the ROS/p53 Pathway to Regulate Ovarian Cancer Cell Proliferation, EMT, and Apoptosis

**Figure 6:**
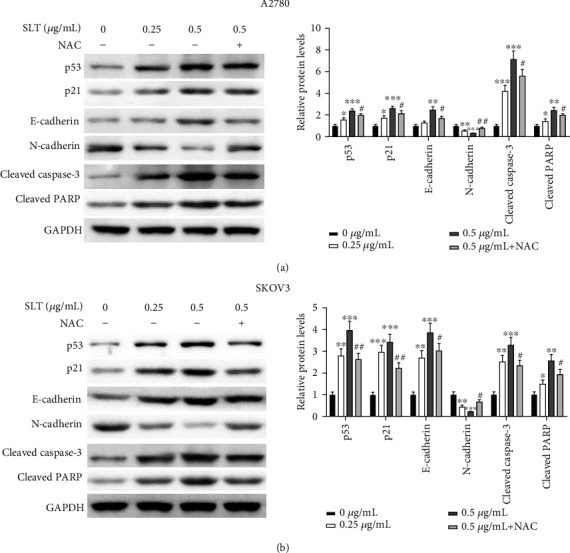
SLT induces the ROS/p53 pathway to regulate ovarian cancer cell proliferation, EMT, and apoptosis. (a) The effects of SLT on the expression levels of p53, p21, E-cadherin, N-cadherin, cleaved caspase-3, and cleaved PARP in A2780 cells were evaluated via western blot assay. (b) The effects of SLT on the expression levels of p53, p21, E-cadherin, N-cadherin, cleaved caspase-3, and cleaved PARP in SKOV3 cells were evaluated via western blot assay. The values were displayed as the mean ± SD. Representative results from three independent experiments were shown (^∗^*P* < 0.05, ^∗∗^*P* < 0.01, and ^∗∗∗^*P* < 0.01, compared with the control group; ^#^*P* < 0.05 and ^##^*P* < 0.01, compared with the 0.5 *μ*g/mL SLT group).

p53 is a tumor suppressor protein which can be activated by a variety of cellular stresses, such as cell death, DNA damage, oxidative stress, and hypoxia [20, 21]. It is reported to be a critical molecule that regulates proliferation, EMT, and apoptosis of cancer cells [22–24]. Hence, we used western blot assay to investigate whether p53 plays important role in SLT-induced ROS-mediated ovarian cancer cell proliferation, EMT, and apoptosis in A2780 and SKOV3 cells. The results indicated that SLT inhibited cell proliferation and EMT and induced cell apoptosis, manifested as increased expression of p21, E-cadherin, cleaved caspase-3, and cleaved PARP, and decreased expression of N-cadherin (Figures 6(a) and 6(b)). Moreover, the expression of p53 was significantly upregulated by SLT (Figures 6(a) and 6(b)), indicating that SLT may affect p53-mediated cell proliferation, EMT, and apoptosis. However, the above effects of SLT partially reversed by pretreatment with NAC (Figures 6(a) and 6(b)). Combined with SLT can promote the production of ROS, p53 can be activated by ROS, so SLT can activate ROS/p53 to regulate ovarian cancer progression, which can be reversed by the effect of inhibiting ROS by NAC treatment. Taken together, these results indicated that the increased generation of ROS induced by SLT intensified p53-mediated signaling cascade to regulate cell proliferation, EMT, and apoptosis.

### 3.7. SLT Regulates the Polarization of M0 Macrophages to Inhibit Ovarian Cancer Progression

**Figure 7:**
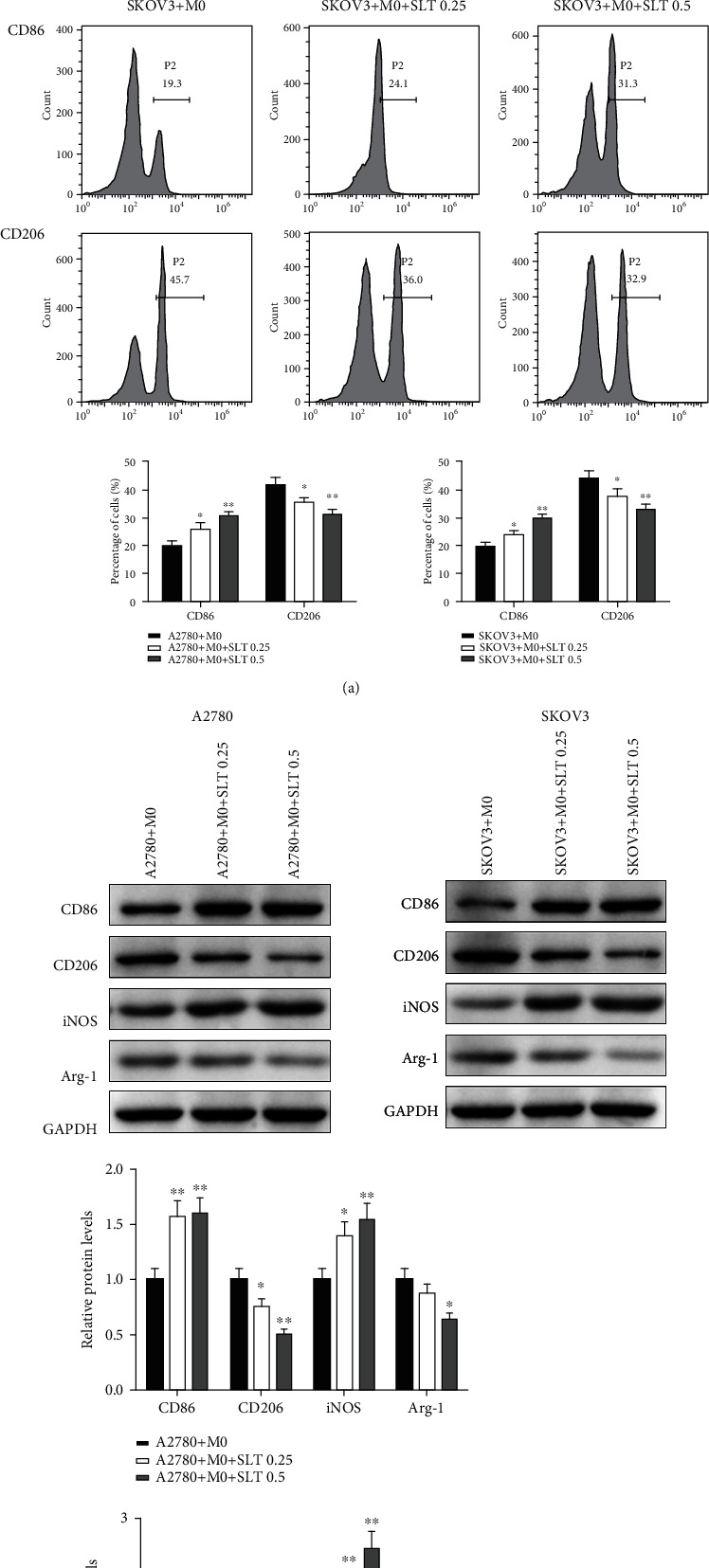
SLT regulates the polarization of M0 macrophages in coculture with ovarian cancer cells. (a) Flow cytometry assay was performed to analyze the M1 and M2 polarization in M0 macrophages, and the percentages of CD86+ and CD206+ cells were detected. (b) The protein expressions of CD86, CD206, iNOS, and Arg-1 were detected using western blotting. The values were displayed as the mean ± SD. Representative results from three independent experiments were shown (^∗^*P* < 0.05, ^∗∗^*P* < 0.01, and ^∗∗∗^*P* < 0.001 compared with the A2780/SKOV3+M0 group).

As the macrophage polarizations are critically involved in the tumor immune microenvironment and thus affect the biological function of tumor cells, we also detect the effect of SLT on the polarization of M0 macrophages cocultured with A2780 and SKOV3 cells, to investigate the regulation potential of SLT on tumor immune microenvironment. Flow cytometry assay was performed to detect the expression of CD86 of M1 macrophages and CD206 of M2 macrophages. As the results indicated in Figure 7(a), compared to the M0 macrophages cocultured with A2780 or SKOV3 cells, the percentage of CD86+ cells was significantly increased in the A2780/SKOV3+M0 macrophages groups treated with SLT (0.25 and 0.5 *μ*g/mL), but the percentage of CD206+ cells was significantly decreased in the A2780/SKOV3+M0 macrophages groups treated with SLT (0.25 and 0.5 *μ*g/mL). In Figure 7(b), the western blot results showed that compared to those in the A2780/SKOV3+M0 macrophages group, the protein expression of CD86 and iNOS was significantly increased in the A2780/SKOV3+M0 macrophages+SLT groups (0.25 and 0.5 *μ*g/mL), and the protein expression of CD206 and Arg-1 was significantly decreased in the A2780/SKOV3+M0 macrophages+SLT groups (0.25 and 0.5 *μ*g/mL). Taken together, these results indicated that in M0 macrophages cocultured with A2780 or SKOV3, SLT could activate the polarization of M0 macrophages to M1 macrophages, but inhibit the polarization of M0 macrophages to M2 macrophages, thus regulate the tumor immune microenvironment and inhibit the ovarian cancer progression.

## 4. Discussion

Ovarian cancer is the most common cancer in women and the second cause of gynecologic cancer death among female worldwide [4]. SLT has been proven to have effective anticancer activity against a variety of cancers, but the antitumor activity against ovarian cancer and its underlying mechanism is rare. This study represented the effect of SLT on ovarian cancer and the potential mechanisms. In the present study, we found that SLT could regulate ovarian cancer cell proliferation, migration, invasion, EMT, and apoptosis through activating the ROS-mediated p53 pathway. The effect on macrophage polarization also showed that SLT activated the M0 macrophages polarized to M1 macrophages, and inhibited to M2 macrophages, to regulate the tumor immune microenvironment of ovarian cancer and thus inhibiting tumor progression.

Our results suggested that SLT plays an important role in inhibiting ovarian tumor progression. Previous study reported that the extract of SLT can inhibit the growth of SMMC-7721 cells and induce apoptosis through the mitochondrial pathway in hepatocellular carcinoma [25]. In human colon adenocarcinoma cells, SLT induced cell cytotoxicity and apoptosis, and regulated the expression of CDK1, p27, p53, cyclin B1, cyclin E, caspase-3, caspase-8, caspase-9, Bcl-2, Bax, and cytochrome C activity [18]. Moreover, sesquiterpenoids extracted from SLT can inhibit the proliferation of breast cancer, colon cancer, gastric cancer cells, etc., by inducing mitochondrial-mediated apoptosis [26]. Considering the role of SLT in other cancers, we found that SLT can inhibit the proliferation and induce the G1 phase arrest in ovarian cancer cells. Consistent with the previous study, we found that SLT significantly reduced the protein levels of CDK4 and Cyclin D1, but increased the protein level of P21. Apoptosis is the process of programmed cell death, which plays a major role in the maintenance of tissue homeostasis and the elimination of tumor cells [27]. Once apoptosis occurs, caspases are activated by proteolytic cleavage and then affect downstream signals, such as PARP [28]. And in this study, SLT was proved to increase the apoptosis rate of ovarian cancer cells through mitochondrial apoptosis pathway with the increased protein levels of cleaved caspase-3, cleaved PARP, Bax, and cytochrome C (in the cytoplasm), while the Bcl-2 level was decreased.

Since most ovarian cancers can only be detected after the cancer has spread to other organs, a promising strategy to fight against this disease is to control the metastasis. EMT is one of the key initiating events of the metastatic cascade, giving tumor cells the ability to invade [29]. EMT and its reverse process mesenchymal transformation to the epithelium (MET) are essential for the metastasis of ovarian cancer; thereby, effective antiovarian cancer treatments can be developed by preventing EMT [30, 31]. MMPs are a family of proteolytic enzymes that include extracellular cell matrix (ECM) modification to accelerate cell migration and cleave cytokines [32]. Increased levels of MMP-2/9 are related to the invasion, metastasis, and poor prognosis of various cancers [33, 34]. A recent study showed that the alkaloid compound solasodine extracted from SLT can induce apoptosis, affect autophagy, and reduce the metastasis of ovarian cancer cells [19]. Therefore, we speculate that SLT can regulate the migration and invasion of ovarian cancer cells. Consistent with our conjecture, SLT was found in this study to inhibit cell migration, invasion, and EMT. Moreover, SLT significantly inhibited the protein levels of MMP2, MMP9, and N-cadherin, and increased the protein level of E-cadherin, indicating that SLT can inhibit EMT in ovarian cancer cells.

**Figure 8:**
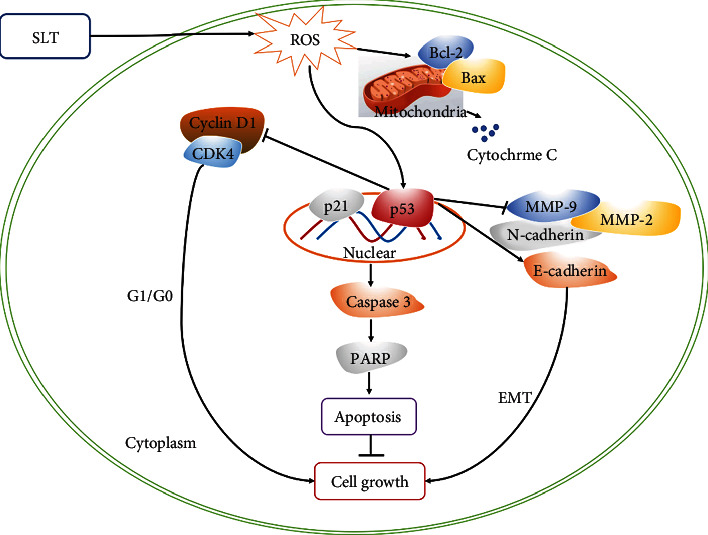
Schematic diagram of the potential molecular mechanism of anticancer effects of SLT on ovarian cancer. →: activation; ⊥: inhibition.

ROS are elevated in response to a variety of stimuli and participate in the regulation of the modulation of cancer cell proliferation, invasion, metastasis, and apoptosis [35, 36]. Many studies have shown that ROS activates the expression of p53 in tumor cells, thereby inhibiting tumor growth [37, 38]. p53 is a well-known tumor suppressor that regulates cell cycle progression, which regulates G0/G1 and G2/M cell cycle checkpoints, as well as downstream p21, and also affects apoptosis and EMT processes [39–41]. Yang et al. found that SLT induced G0/G1 phase arrest and triggered apoptosis via p53 activation in the WEHI-3 murine leukemia cells [42]. Therefore, we speculated that the p53 signaling pathway may be a key mechanism regulating the anticancer effects of SLT in ovarian cancer cells. Consistently, SLT was proved to promote ROS accumulation, inhibit cell proliferation, EMT, and induce apoptosis, and the mechanism might be associated with the p53 pathway, whereas the effects can be reversed by inhibiting ROS by NAC treatment. A schematic diagram depicting the hypothetical mechanism required for SLT treatment is illustrated in Figure 8. Taken together, the results suggested that the ROS/p53 pathway might be related to SLT-regulated proliferation, invasion, and apoptosis in ovarian cancer cells.

Tumor-associated macrophages are one of the most abundant and significant innate immune cells in tumor microenvironment; M1 macrophages suppress cancer progression, while M2 macrophages promote it [43]. In *in vitro* simulated tumor microenvironment, study shows that cocultured ovarian cancer cells polarized macrophages to the M2 phenotype, and M2 macrophages enhanced the proliferation, invasion, and migration, and inhibited the apoptosis of ovarian cancer cells, thus playing a stimulation role in ovarian cancer cells [44]. And in ovarian cancer patients, increased overall or intraislet M1/M2 TAM ratios were positively correlated with an improved 5-year prognosis [45]. In this study, we also found that in ovarian cancer cells cocultured with M0 macrophages, that SLT activated the polarization of M0 macrophages to M1 macrophages and inhibited the polarization to M2 macrophages, with the increased percentage of CD86+ cells and decreased percentage of CD206+ cells were detected. It indicated the important role of macrophage polarization as well as the immunoregulation potential of SLT in tumor microenvironment of ovarian cancer cells. Previous study also reported the immunomodulatory activity of SLT extract in tumor, by remarkably promoting splenocyte proliferation, NK cell, and CTL activity in S180 sarcoma tumor-bearing mice, indicated SLT could act as antitumor agent with immunomodulatory activity [46].

In conclusion, our data showed that SLT can inhibit the proliferation, induce apoptosis, inhibit migration, invasion, and EMT, and also regulate macrophage polarization to regulate tumor immune microenvironment in ovarian cancer cells. The results also demonstrate that the mechanism might be achieved by regulating the ROS-mediated p53 signaling pathway. Taken together, these findings may provide insights to better understand the potential mechanisms of the anticancer effects of SLT and help to discover alternative treatment strategies for ovarian cancer.

## 5. Conclusion

In summary, this study illustrated the anticancer effects of SLT on ovarian cancer cells, suggesting that SLT may have the potential to provide basic evidence for the discovery of antiovarian cancer agents.

## Data Availability

All data generated or analyzed during this study are included in this article.
